# Supplementary material for: Development and Validation of a Meta-Instrument for the Assessment of Functional Capacity, the Risk of Falls and Pressure Injuries in Adult Hospitalization Units (VALENF Instrument) (Part II)
Source: Int J Environ Res Public Health. 2023 Mar 12;20(6):5003. doi: 10.3390/ijerph20065003 (PMC10049057; doi:10.3390/ijerph20065003)

Supplementary Material

Path Diagrams of Confirmatory Factor Analysis

Path Diagram of Confirmatory Factor Analysis considering the seven items of VALENF Instrument.

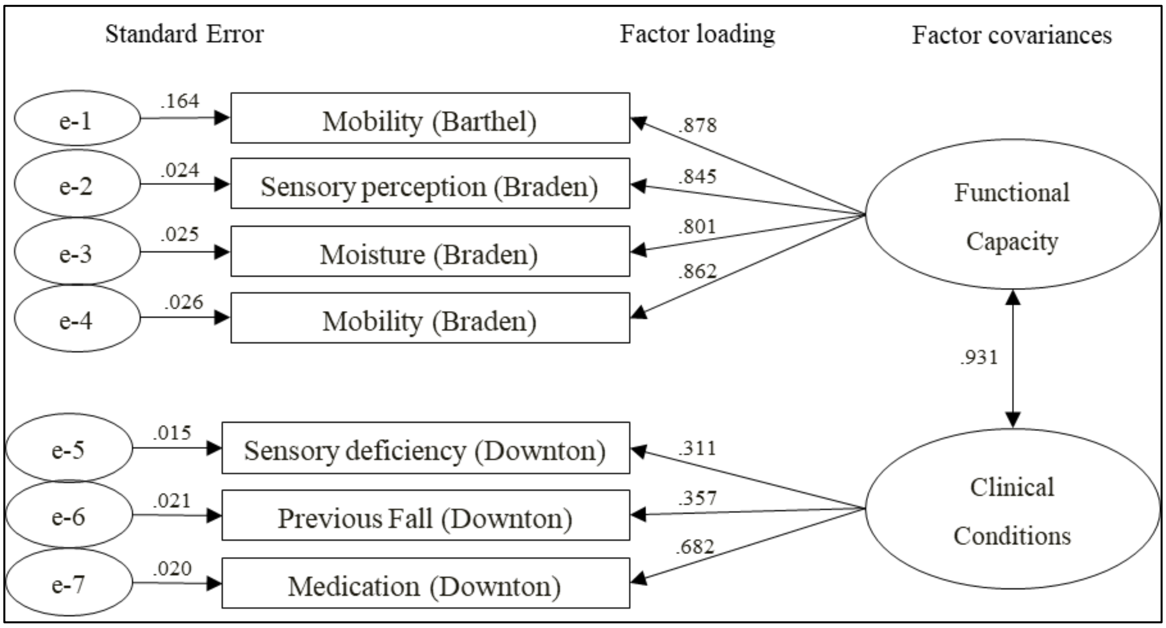

Path Diagram of Confirmatory Factor Analysis considering the items with predictive capacity on the Barthel index.

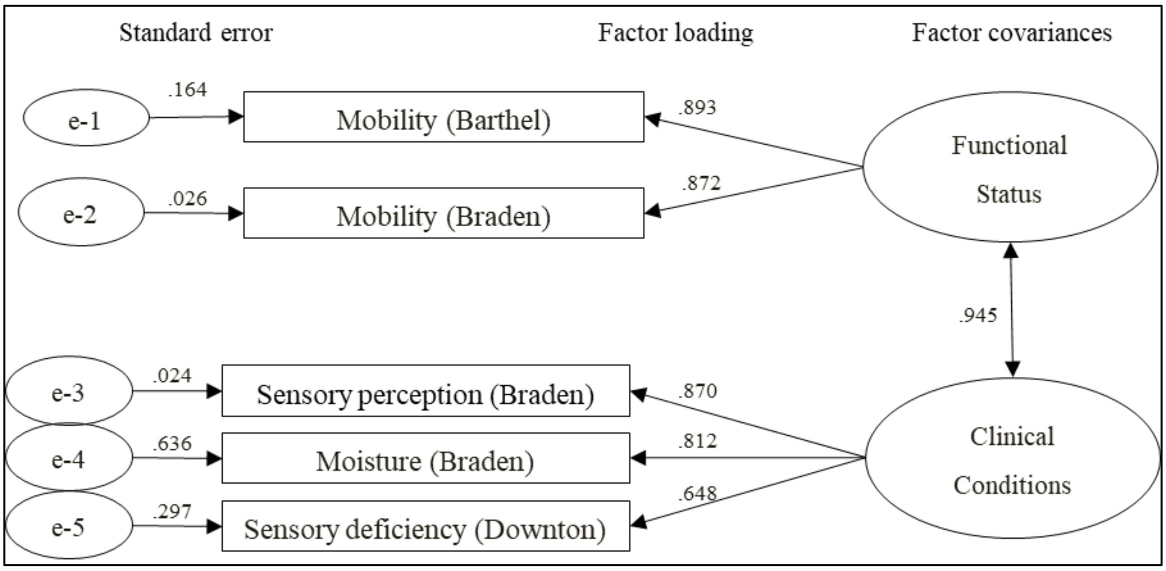

Path Diagram of Confirmatory Factor Analysis considering the items with predictive capacity on the Braden index.

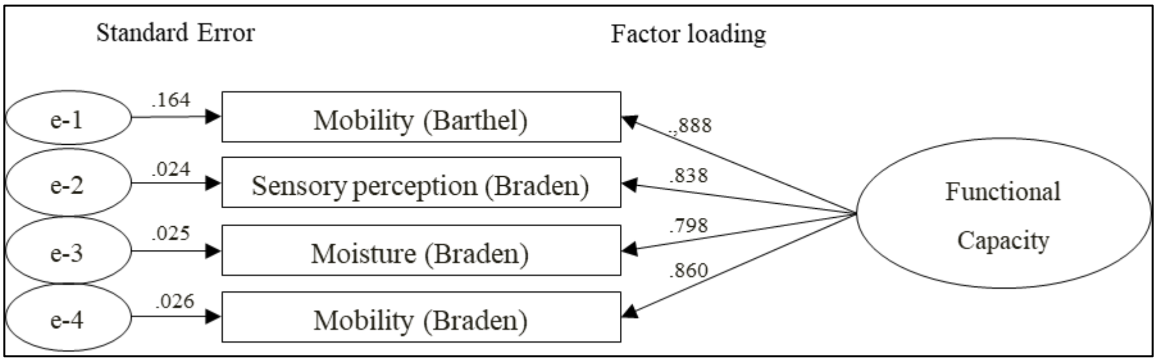

Path Diagram of Confirmatory Factor Analysis considering the items with predictive capacity on the Downton index.

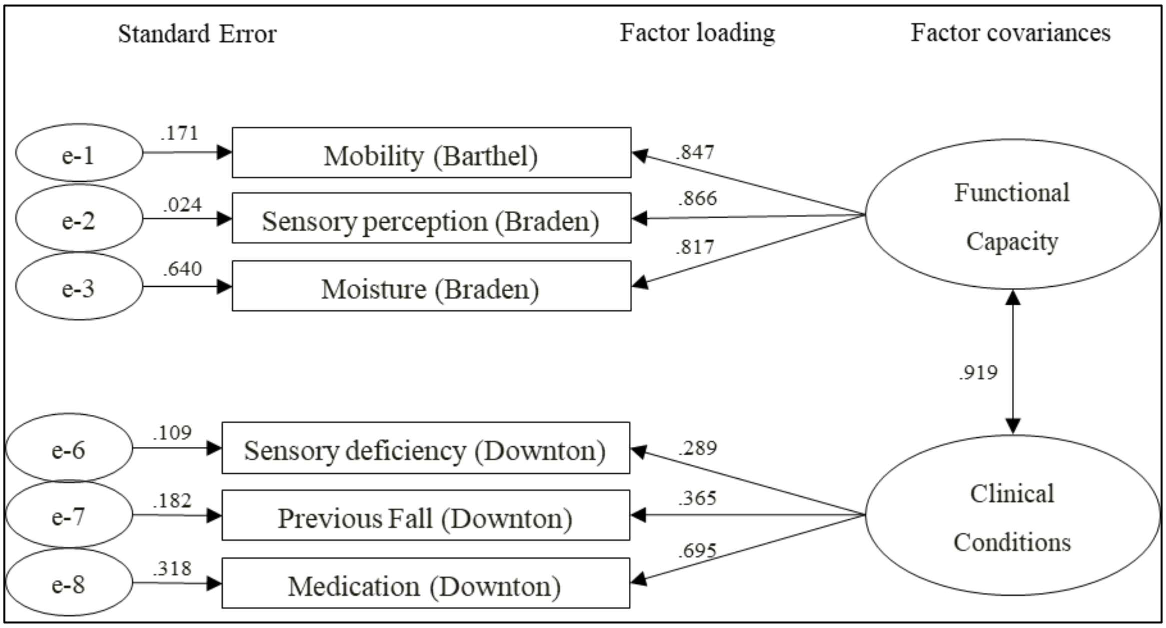

Supplement: Supplementary file 1 [file ijerph-20-05003-s001.zip › ijerph-2233553-supplementary.pdf]
